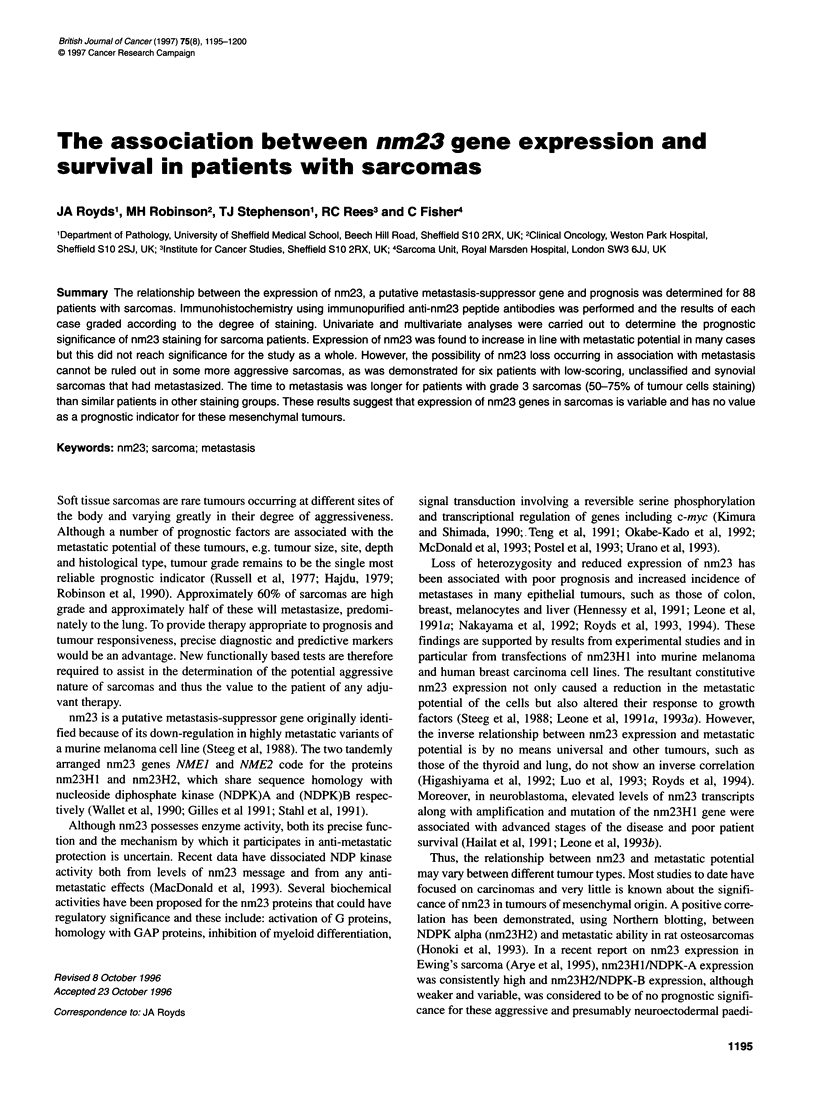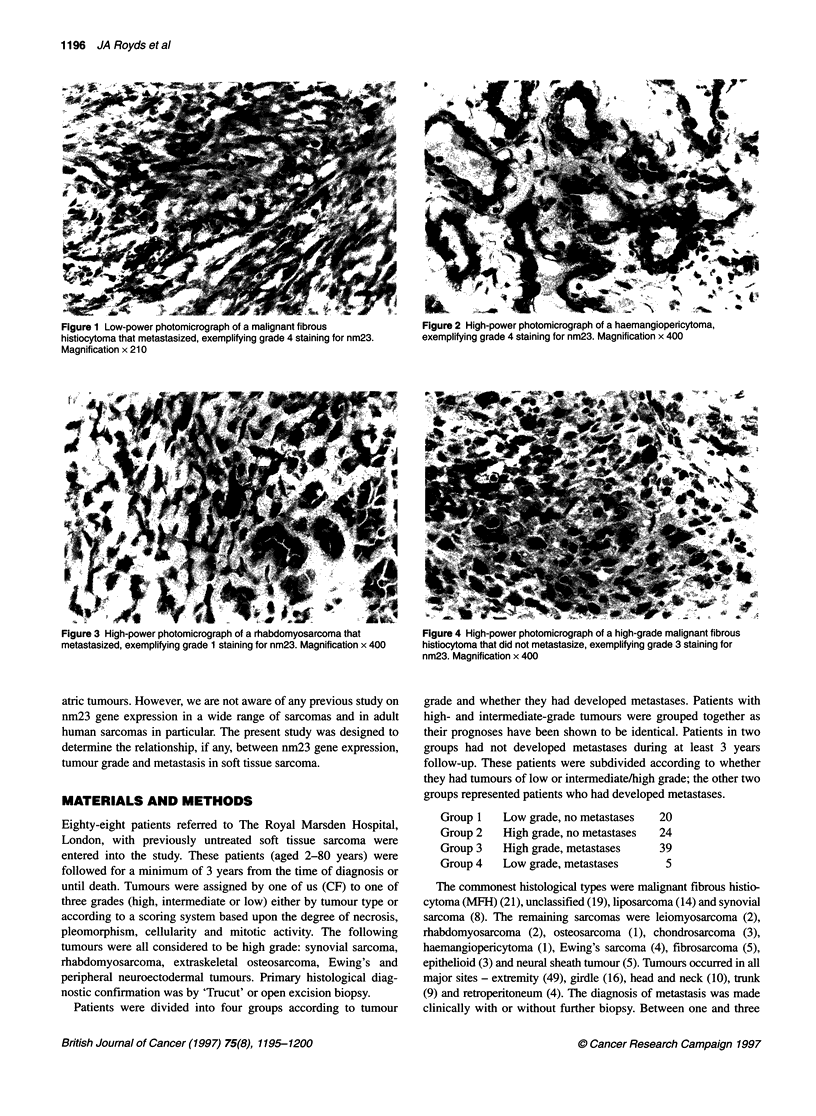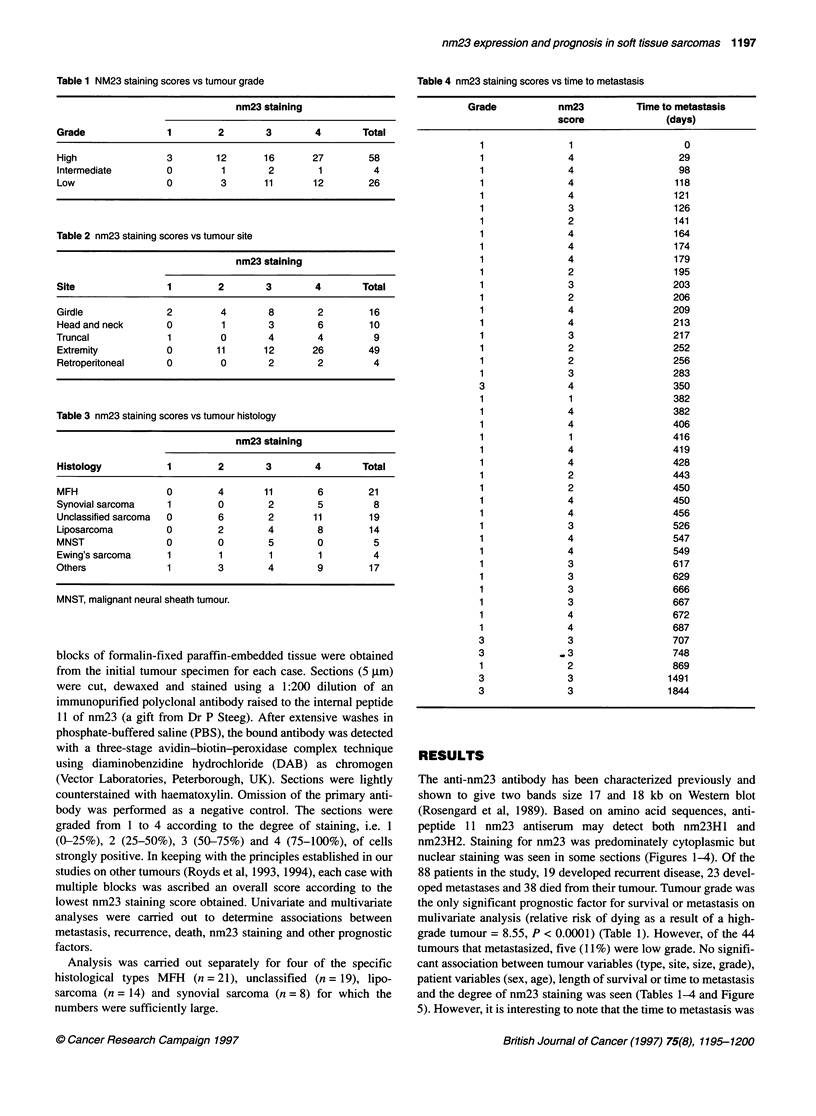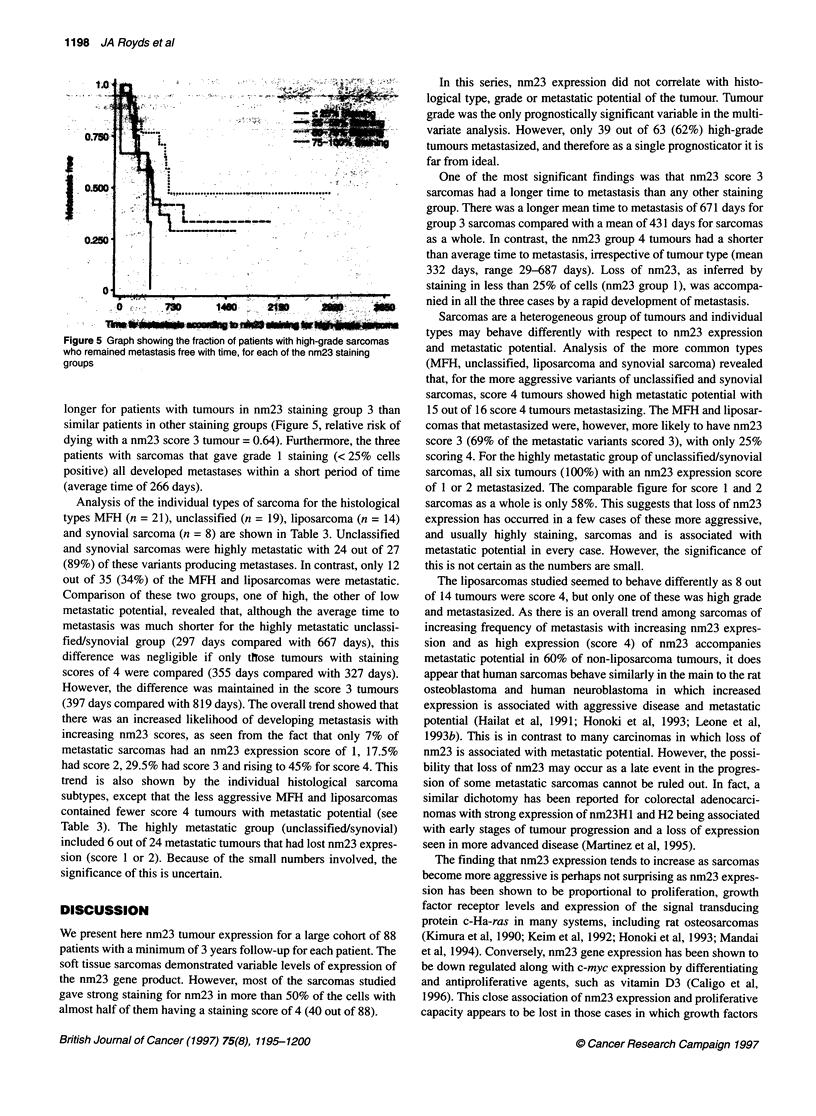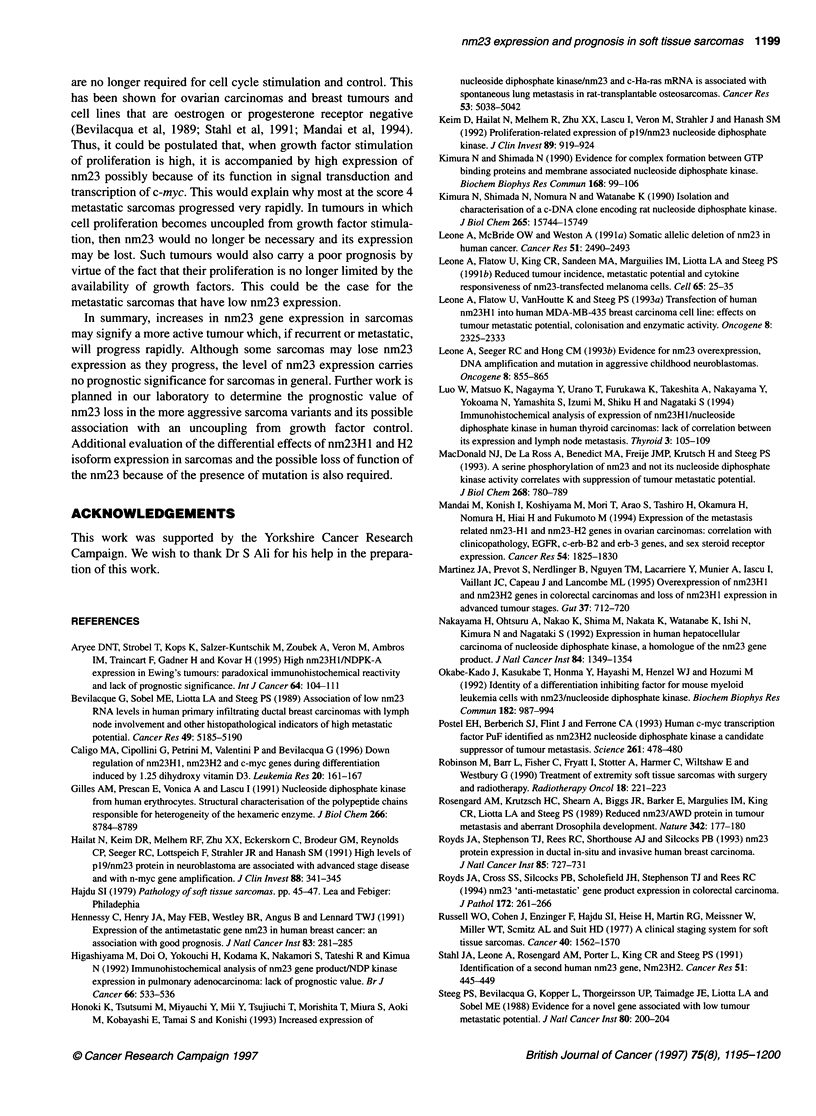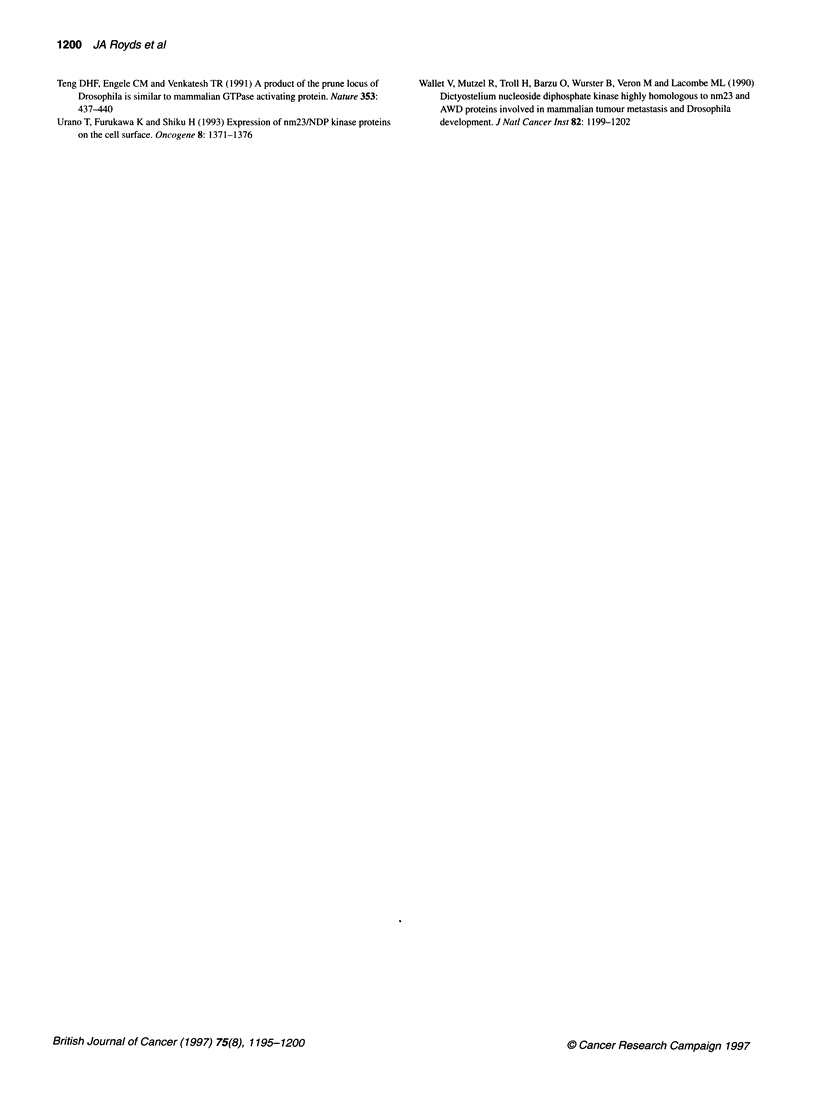# The association between nm23 gene expression and survival in patients with sarcomas.

**DOI:** 10.1038/bjc.1997.204

**Published:** 1997

**Authors:** J. A. Royds, M. H. Robinson, T. J. Stephenson, R. C. Rees, C. Fisher

**Affiliations:** Department of Pathology, University of Sheffield Medical School, UK.

## Abstract

**Images:**


British Journal of Cancer (1997) 75(8), 1195–1200
© 1997 Cancer Research Campaign

### The association between *nm23* gene expression and survival in patients with sarcomas

**JA Royds[1], MH Robinson[2], TJ Stephenson[1], RC Rees[3] and C Fisher[4]**

[1]Department of Pathology, University of Sheffield Medical School, Beech Hill Road, Sheffield S10 2RX, UK; [2]Clinical Oncology, Weston Park Hospital, Sheffield S10 2SJ, UK; [3]Institute for Cancer Studies, Sheffield S10 2RX, UK; [4]Sarcoma Unit, Royal Marsden Hospital, London SW3 6JJ, UK

**Summary** The relationship between the expression of nm23, a putative metastasis-suppressor gene and prognosis was determined for 88 patients with sarcomas. Immunohistochemistry using immunopurified anti-nm23 peptide antibodies was performed and the results of each case graded according to the degree of staining. Univariate and multivariate analyses were carried out to determine the prognostic significance of nm23 staining for sarcoma patients. Expression of nm23 was found to increase in line with metastatic potential in many cases but this did not reach significance for the study as a whole. However, the possibility of nm23 loss occurring in association with metastasis cannot be ruled out in some more aggressive sarcomas, as was demonstrated for six patients with low-scoring, unclassified and synovial sarcomas that had metastasized. The time to metastasis was longer for patients with grade 3 sarcomas (50–75% of tumour cells staining) than similar patients in other staining groups. These results suggest that expression of nm23 genes in sarcomas is variable and has no value as a prognostic indicator for these mesenchymal tumours.

**Keywords:** nm23; sarcoma; metastasis

Soft tissue sarcomas are rare tumours occurring at different sites of the body and varying greatly in their degree of aggressiveness. Although a number of prognostic factors are associated with the metastatic potential of these tumours, e.g. tumour size, site, depth and histological type, tumour grade remains to be the single most reliable prognostic indicator (Russell et al, 1977; Hajdu, 1979; Robinson et al, 1990). Approximately 60% of sarcomas are high grade and approximately half of these will metastasize, predominately to the lung. To provide therapy appropriate to prognosis and tumour responsiveness, precise diagnostic and predictive markers would be an advantage. New functionally based tests are therefore required to assist in the determination of the potential aggressive nature of sarcomas and thus the value to the patient of any adjuvant therapy.

nm23 is a putative metastasis-suppressor gene originally identified because of its down-regulation in highly metastatic variants of a murine melanoma cell line (Steeg et al, 1988). The two tandemly arranged nm23 genes *NME1* and *NME2* code for the proteins nm23H1 and nm23H2, which share sequence homology with nucleoside diphosphate kinase (NDPK)A and (NDPK)B respectively (Wallet et al, 1990; Gilles et al 1991; Stahl et al, 1991).

Although nm23 possesses enzyme activity, both its precise function and the mechanism by which it participates in anti-metastatic protection is uncertain. Recent data have dissociated NDP kinase activity both from levels of nm23 message and from any anti-metastatic effects (MacDonald et al, 1993). Several biochemical activities have been proposed for the nm23 proteins that could have regulatory significance and these include: activation of G proteins, homology with GAP proteins, inhibition of myeloid differentiation, signal transduction involving a reversible serine phosphorylation and transcriptional regulation of genes including c-*myc* (Kimura and Shimada, 1990; Teng et al, 1991; Okabe-Kado et al, 1992; McDonald et al, 1993; Postel et al, 1993; Urano et al, 1993).

Loss of heterozygosity and reduced expression of nm23 has been associated with poor prognosis and increased incidence of metastases in many epithelial tumours, such as those of colon, breast, melanocytes and liver (Hennessy et al, 1991; Leone et al, 1991*a*; Nakayama et al, 1992; Royds et al, 1993, 1994). These findings are supported by results from experimental studies and in particular from transfections of nm23H1 into murine melanoma and human breast carcinoma cell lines. The resultant constitutive nm23 expression not only caused a reduction in the metastatic potential of the cells but also altered their response to growth factors (Steeg et al, 1988; Leone et al, 1991*a*, 1993*a*). However, the inverse relationship between nm23 expression and metastatic potential is by no means universal and other tumours, such as those of the thyroid and lung, do not show an inverse correlation (Higashiyama et al, 1992; Luo et al, 1993; Royds et al, 1994). Moreover, in neuroblastoma, elevated levels of nm23 transcripts along with amplification and mutation of the nm23H1 gene were associated with advanced stages of the disease and poor patient survival (Hailat et al, 1991; Leone et al, 1993*b*).

Thus, the relationship between nm23 and metastatic potential may vary between different tumour types. Most studies to date have focused on carcinomas and very little is known about the significance of nm23 in tumours of mesenchymal origin. A positive correlation has been demonstrated, using Northern blotting, between NDPK alpha (nm23H2) and metastatic ability in rat osteosarcomas (Honoki et al, 1993). In a recent report on nm23 expression in Ewing's sarcoma (Arye et al, 1995), nm23H1/NDPK-A expression was consistently high and nm23H2/NDPK-B expression, although weaker and variable, was considered to be of no prognostic significance for these aggressive and presumably neuroectodermal paedi-

*Revised 8 October 1996*
*Accepted 23 October 1996*

*Correspondence to:* JA Royds

**Figure 1** Low-power photomicrograph of a malignant fibrous histiocytoma that metastasized, exemplifying grade 4 staining for nm23. Magnification × 210

**Figure 2** High-power photomicrograph of a haemangiopericytoma, exemplifying grade 4 staining for nm23. Magnification × 400

**Figure 3** High-power photomicrograph of a rhabdomyosarcoma that metastasized, exemplifying grade 1 staining for nm23. Magnification × 400

**Figure 4** High-power photomicrograph of a high-grade malignant fibrous histiocytoma that did not metastasize, exemplifying grade 3 staining for nm23. Magnification × 400

atric tumours. However, we are not aware of any previous study on nm23 gene expression in a wide range of sarcomas and in adult human sarcomas in particular. The present study was designed to determine the relationship, if any, between nm23 gene expression, tumour grade and metastasis in soft tissue sarcoma.

### MATERIALS AND METHODS

Eighty-eight patients referred to The Royal Marsden Hospital, London, with previously untreated soft tissue sarcoma were entered into the study. These patients (aged 2–80 years) were followed for a minimum of 3 years from the time of diagnosis or until death. Tumours were assigned by one of us (CF) to one of three grades (high, intermediate or low) either by tumour type or according to a scoring system based upon the degree of necrosis, pleomorphism, cellularity and mitotic activity. The following tumours were all considered to be high grade: synovial sarcoma, rhabdomyosarcoma, extraskeletal osteosarcoma, Ewing's and peripheral neuroectodermal tumours. Primary histological diagnostic confirmation was by 'Trucut' or open excision biopsy.

Patients were divided into four groups according to tumour grade and whether they had developed metastases. Patients with high- and intermediate-grade tumours were grouped together as their prognoses have been shown to be identical. Patients in two groups had not developed metastases during at least 3 years follow-up. These patients were subdivided according to whether they had tumours of low or intermediate/high grade; the other two groups represented patients who had developed metastases.

| | | |
|---|---|---|
| Group 1 | Low grade, no metastases | 20 |
| Group 2 | High grade, no metastases | 24 |
| Group 3 | High grade, metastases | 39 |
| Group 4 | Low grade, metastases | 5 |

The commonest histological types were malignant fibrous histiocytoma (MFH) (21), unclassified (19), liposarcoma (14) and synovial sarcoma (8). The remaining sarcomas were leiomyosarcoma (2), rhabdomyosarcoma (2), osteosarcoma (1), chondrosarcoma (3), haemangiopericytoma (1), Ewing's sarcoma (4), fibrosarcoma (5), epithelioid (3) and neural sheath tumour (5). Tumours occurred in all major sites – extremity (49), girdle (16), head and neck (10), trunk (9) and retroperitoneum (4). The diagnosis of metastasis was made clinically with or without further biopsy. Between one and three

*© Cancer Research Campaign 1997*

**Table 1** NM23 staining scores vs tumour grade

| Grade | nm23 staining 1 | 2 | 3 | 4 | Total |
|---|---|---|---|---|---|
| High | 3 | 12 | 16 | 27 | 58 |
| Intermediate | 0 | 1 | 2 | 1 | 4 |
| Low | 0 | 3 | 11 | 12 | 26 |

**Table 2** nm23 staining scores vs tumour site

| Site | nm23 staining 1 | 2 | 3 | 4 | Total |
|---|---|---|---|---|---|
| Girdle | 2 | 4 | 8 | 2 | 16 |
| Head and neck | 0 | 1 | 3 | 6 | 10 |
| Truncal | 1 | 0 | 4 | 4 | 9 |
| Extremity | 0 | 11 | 12 | 26 | 49 |
| Retroperitoneal | 0 | 0 | 2 | 2 | 4 |

**Table 3** nm23 staining scores vs tumour histology

| Histology | nm23 staining 1 | 2 | 3 | 4 | Total |
|---|---|---|---|---|---|
| MFH | 0 | 4 | 11 | 6 | 21 |
| Synovial sarcoma | 1 | 0 | 2 | 5 | 8 |
| Unclassified sarcoma | 0 | 6 | 2 | 11 | 19 |
| Liposarcoma | 0 | 2 | 4 | 8 | 14 |
| MNST | 0 | 0 | 5 | 0 | 5 |
| Ewing's sarcoma | 1 | 1 | 1 | 1 | 4 |
| Others | 1 | 3 | 4 | 9 | 17 |

MNST, malignant neural sheath tumour.

blocks of formalin-fixed paraffin-embedded tissue were obtained from the initial tumour specimen for each case. Sections (5 μm) were cut, dewaxed and stained using a 1:200 dilution of an immunopurified polyclonal antibody raised to the internal peptide 11 of nm23 (a gift from Dr P Steeg). After extensive washes in phosphate-buffered saline (PBS), the bound antibody was detected with a three-stage avidin–biotin–peroxidase complex technique using diaminobenzidine hydrochloride (DAB) as chromogen (Vector Laboratories, Peterborough, UK). Sections were lightly counterstained with haematoxylin. Omission of the primary antibody was performed as a negative control. The sections were graded from 1 to 4 according to the degree of staining, i.e. 1 (0–25%), 2 (25–50%), 3 (50–75%) and 4 (75–100%), of cells strongly positive. In keeping with the principles established in our studies on other tumours (Royds et al, 1993, 1994), each case with multiple blocks was ascribed an overall score according to the lowest nm23 staining score obtained. Univariate and multivariate analyses were carried out to determine associations between metastasis, recurrence, death, nm23 staining and other prognostic factors.

Analysis was carried out separately for four of the specific histological types MFH ($n = 21$), unclassified ($n = 19$), liposarcoma ($n = 14$) and synovial sarcoma ($n = 8$) for which the numbers were sufficiently large.

**Table 4** nm23 staining scores vs time to metastasis

| Grade | nm23 score | Time to metastasis (days) |
|---|---|---|
| 1 | 1 | 0 |
| 1 | 4 | 29 |
| 1 | 4 | 98 |
| 1 | 4 | 118 |
| 1 | 4 | 121 |
| 1 | 3 | 126 |
| 1 | 2 | 141 |
| 1 | 4 | 164 |
| 1 | 4 | 174 |
| 1 | 4 | 179 |
| 1 | 2 | 195 |
| 1 | 3 | 203 |
| 1 | 2 | 206 |
| 1 | 4 | 209 |
| 1 | 4 | 213 |
| 1 | 3 | 217 |
| 1 | 2 | 252 |
| 1 | 2 | 256 |
| 1 | 3 | 283 |
| 3 | 4 | 350 |
| 1 | 1 | 382 |
| 1 | 4 | 382 |
| 1 | 4 | 406 |
| 1 | 1 | 416 |
| 1 | 4 | 419 |
| 1 | 4 | 428 |
| 1 | 2 | 443 |
| 1 | 2 | 450 |
| 1 | 4 | 450 |
| 1 | 4 | 456 |
| 1 | 3 | 526 |
| 1 | 4 | 547 |
| 1 | 4 | 549 |
| 1 | 3 | 617 |
| 1 | 3 | 629 |
| 1 | 3 | 666 |
| 1 | 3 | 667 |
| 1 | 4 | 672 |
| 1 | 4 | 687 |
| 3 | 3 | 707 |
| 3 | 3 | 748 |
| 1 | 2 | 869 |
| 3 | 3 | 1491 |
| 3 | 3 | 1844 |

### RESULTS

The anti-nm23 antibody has been characterized previously and shown to give two bands size 17 and 18 kb on Western blot (Rosengard et al, 1989). Based on amino acid sequences, anti-peptide 11 nm23 antiserum may detect both nm23H1 and nm23H2. Staining for nm23 was predominately cytoplasmic but nuclear staining was seen in some sections (Figures 1–4). Of the 88 patients in the study, 19 developed recurrent disease, 23 developed metastases and 38 died from their tumour. Tumour grade was the only significant prognostic factor for survival or metastasis on mulivariate analysis (relative risk of dying as a result of a high-grade tumour = 8.55, $P < 0.0001$) (Table 1). However, of the 44 tumours that metastasized, five (11%) were low grade. No significant association between tumour variables (type, site, size, grade), patient variables (sex, age), length of survival or time to metastasis and the degree of nm23 staining was seen (Tables 1–4 and Figure 5). However, it is interesting to note that the time to metastasis was

© Cancer Research Campaign 1997

**Figure 5** Graph showing the fraction of patients with high-grade sarcomas who remained metastasis free with time, for each of the nm23 staining groups longer for patients with tumours in nm23 staining group 3 than similar patients in other staining groups (Figure 5, relative risk of dying with a nm23 score 3 tumour = 0.64). Furthermore, the three patients with sarcomas that gave grade 1 staining (< 25% cells positive) all developed metastases within a short period of time (average time of 266 days).

Analysis of the individual types of sarcoma for the histological types MFH ($n$ = 21), unclassified ($n$ = 19), liposarcoma ($n$ = 14) and synovial sarcoma ($n$ = 8) are shown in Table 3. Unclassified and synovial sarcomas were highly metastatic with 24 out of 27 (89%) of these variants producing metastases. In contrast, only 12 out of 35 (34%) of the MFH and liposarcomas were metastatic. Comparison of these two groups, one of high, the other of low metastatic potential, revealed that, although the average time to metastasis was much shorter for the highly metastatic unclassified/synovial group (297 days compared with 667 days), this difference was negligible if only those tumours with staining scores of 4 were compared (355 days compared with 327 days). However, the difference was maintained in the score 3 tumours (397 days compared with 819 days). The overall trend showed that there was an increased likelihood of developing metastasis with increasing nm23 scores, as seen from the fact that only 7% of metastatic sarcomas had an nm23 expression score of 1, 17.5% had score 2, 29.5% had score 3 and rising to 45% for score 4. This trend is also shown by the individual histological sarcoma subtypes, except that the less aggressive MFH and liposarcomas contained fewer score 4 tumours with metastatic potential (see Table 3). The highly metastatic group (unclassified/synovial) included 6 out of 24 metastatic tumours that had lost nm23 expression (score 1 or 2). Because of the small numbers involved, the significance of this is uncertain.

### DISCUSSION

We present here nm23 tumour expression for a large cohort of 88 patients with a minimum of 3 years follow-up for each patient. The soft tissue sarcomas demonstrated variable levels of expression of the nm23 gene product. However, most of the sarcomas studied gave strong staining for nm23 in more than 50% of the cells with almost half of them having a staining score of 4 (40 out of 88).

In this series, nm23 expression did not correlate with histological type, grade or metastatic potential of the tumour. Tumour grade was the only prognostically significant variable in the multivariate analysis. However, only 39 out of 63 (62%) high-grade tumours metastasized, and therefore as a single prognosticator it is far from ideal.

One of the most significant findings was that nm23 score 3 sarcomas had a longer time to metastasis than any other staining group. There was a longer mean time to metastasis of 671 days for group 3 sarcomas compared with a mean of 431 days for sarcomas as a whole. In contrast, the nm23 group 4 tumours had a shorter than average time to metastasis, irrespective of tumour type (mean 332 days, range 29–687 days). Loss of nm23, as inferred by staining in less than 25% of cells (nm23 group 1), was accompanied in all the three cases by a rapid development of metastasis.

Sarcomas are a heterogeneous group of tumours and individual types may behave differently with respect to nm23 expression and metastatic potential. Analysis of the more common types (MFH, unclassified, liposarcoma and synovial sarcoma) revealed that, for the more aggressive variants of unclassified and synovial sarcomas, score 4 tumours showed high metastatic potential with 15 out of 16 score 4 tumours metastasizing. The MFH and liposarcomas that metastasized were, however, more likely to have nm23 score 3 (69% of the metastatic variants scored 3), with only 25% scoring 4. For the highly metastatic group of unclassified/synovial sarcomas, all six tumours (100%) with an nm23 expression score of 1 or 2 metastasized. The comparable figure for score 1 and 2 sarcomas as a whole is only 58%. This suggests that loss of nm23 expression has occurred in a few cases of these more aggressive, and usually highly staining, sarcomas and is associated with metastatic potential in every case. However, the significance of this is not certain as the numbers are small.

The liposarcomas studied seemed to behave differently as 8 out of 14 tumours were score 4, but only one of these was high grade and metastasized. As there is an overall trend among sarcomas of increasing frequency of metastasis with increasing nm23 expression and as high expression (score 4) of nm23 accompanies metastatic potential in 60% of non-liposarcoma tumours, it does appear that human sarcomas behave similarly in the main to the rat osteoblastoma and human neuroblastoma in which increased expression is associated with aggressive disease and metastatic potential (Hailat et al, 1991; Honoki et al, 1993; Leone et al, 1993*b*). This is in contrast to many carcinomas in which loss of nm23 is associated with metastatic potential. However, the possibility that loss of nm23 may occur as a late event in the progression of some metastatic sarcomas cannot be ruled out. In fact, a similar dichotomy has been reported for colorectal adenocarcinomas with strong expression of nm23H1 and H2 being associated with early stages of tumour progression and a loss of expression seen in more advanced disease (Martinez et al, 1995).

The finding that nm23 expression tends to increase as sarcomas become more aggressive is perhaps not surprising as nm23 expression has been shown to be proportional to proliferation, growth factor receptor levels and expression of the signal transducing protein c-Ha-*ras* in many systems, including rat osteosarcomas (Kimura et al, 1990; Keim et al, 1992; Honoki et al, 1993; Mandai et al, 1994). Conversely, nm23 gene expression has been shown to be down regulated along with c-*myc* expression by differentiating and antiproliferative agents, such as vitamin D3 (Caligo et al, 1996). This close association of nm23 expression and proliferative capacity appears to be lost in those cases in which growth factors

© Cancer Research Campaign 1997

are no longer required for cell cycle stimulation and control. This has been shown for ovarian carcinomas and breast tumours and cell lines that are oestrogen or progesterone receptor negative (Bevilacqua et al, 1989; Stahl et al, 1991; Mandai et al, 1994). Thus, it could be postulated that, when growth factor stimulation of proliferation is high, it is accompanied by high expression of nm23 possibly because of its function in signal transduction and transcription of c-*myc*. This would explain why most at the score 4 metastatic sarcomas progressed very rapidly. In tumours in which cell proliferation becomes uncoupled from growth factor stimulation, then nm23 would no longer be necessary and its expression may be lost. Such tumours would also carry a poor prognosis by virtue of the fact that their proliferation is no longer limited by the availability of growth factors. This could be the case for the metastatic sarcomas that have low nm23 expression.

In summary, increases in nm23 gene expression in sarcomas may signify a more active tumour which, if recurrent or metastatic, will progress rapidly. Although some sarcomas may lose nm23 expression as they progress, the level of nm23 expression carries no prognostic significance for sarcomas in general. Further work is planned in our laboratory to determine the prognostic value of nm23 loss in the more aggressive sarcoma variants and its possible association with an uncoupling from growth factor control. Additional evaluation of the differential effects of nm23H1 and H2 isoform expression in sarcomas and the possible loss of function of the nm23 because of the presence of mutation is also required.

### ACKNOWLEDGEMENTS

This work was supported by the Yorkshire Cancer Research Campaign. We wish to thank Dr S Ali for his help in the preparation of this work.

### REFERENCES

Aryee DNT, Strobel T, Kops K, Salzer-Kuntschik M, Zoubek A, Veron M, Ambros IM, Traincart F, Gadner H and Kovar H (1995) High nm23H1/NDPK-A expression in Ewing's tumours: paradoxical immunohistochemical reactivity and lack of prognostic significance. *Int J Cancer* **64**: 104–111

Bevilacque G, Sobel ME, Liotta LA and Steeg PS (1989) Association of low nm23 RNA levels in human primary infiltrating ductal breast carcinomas with lymph node involvement and other histopathological indicators of high metastatic potential. *Cancer Res* **49**: 5185–5190

Caligo MA, Cipollini G, Petrini M, Valentini P and Bevilacqua G (1996) Down regulation of nm23H1, nm23H2 and c-myc genes during differentiation induced by 1.25 dihydroxy vitamin D3. *Leukemia Res* **20**: 161–167

Gilles AM, Prescan E, Vonica A and Lascu I (1991) Nucleoside diphosphate kinase from human erythrocytes. Structural characterisation of the polypeptide chains responsible for heterogeneity of the hexameric enzyme. *J Biol Chem* **266**: 8784–8789

Hailat N, Keim DR, Melhem RF, Zhu XX, Eckerskorn C, Brodeur GM, Reynolds CP, Seeger RC, Lottspeich F, Strahler JR and Hanash SM (1991) High levels of p19/nm23 protein in neuroblastoma are associated with advanced stage disease and with n-myc gene amplification. *J Clin Invest* **88**: 341–345

Hajdu SI (1979) *Pathology of soft tissue sarcomas*. pp. 45–47. Lea and Febiger: Philadephia

Hennessy C, Henry JA, May FEB, Westley BR, Angus B and Lennard TWJ (1991) Expression of the antimetastatic gene nm23 in human breast cancer: an association with good prognosis. *J Natl Cancer Inst* **83**: 281–285

Higashiyama M, Doi O, Yokouchi H, Kodama K, Nakamori S, Tateshi R and Kimua N (1992) Immunohistochemical analysis of nm23 gene product/NDP kinase expression in pulmonary adenocarcinoma: lack of prognostic value. *Br J Cancer* **66**: 533–536

Honoki K, Tsutsumi M, Miyauchi Y, Mii Y, Tsujiuchi T, Morishita T, Miura S, Aoki M, Kobayashi E, Tamai S and Konishi (1993) Increased expression of nucleoside diphosphate kinase/nm23 and c-Ha-ras mRNA is associated with spontaneous lung metastasis in rat-transplantable osteosarcomas. *Cancer Res* **53**: 5038–5042

Keim D, Hailat N, Melhem R, Zhu XX, Lascu I, Veron M, Strahler J and Hanash SM (1992) Proliferation-related expression of p19/nm23 nucleoside diphosphate kinase. *J Clin Invest* **89**: 919–924

Kimura N and Shimada N (1990) Evidence for complex formation between GTP binding proteins and membrane associated nucleoside diphosphate kinase. *Biochem Biophys Res Commun* **168**: 99–106

Kimura N, Shimada N, Nomura N and Watanabe K (1990) Isolation and characterisation of a c-DNA clone encoding rat nucleoside diphosphate kinase. *J Biol Chem* **265**: 15744–15749

Leone A, McBride OW and Weston A (1991*a*) Somatic allelic deletion of nm23 in human cancer. *Cancer Res* **51**: 2490–2493

Leone A, Flatow U, King CR, Sandeen MA, Marguilies IM, Liotta LA and Steeg PS (1991*b*) Reduced tumour incidence, metastatic potential and cytokine responsiveness of nm23-transfected melanoma cells. *Cell* **65**: 25–35

Leone A, Flatow U, VanHoutte K and Steeg PS (1993*a*) Transfection of human nm23H1 into human MDA-MB-435 breast carcinoma cell line: effects on tumour metastatic potential, colonisation and enzymatic activity. *Oncogene* **8**: 2325–2333

Leone A, Seeger RC and Hong CM (1993*b*) Evidence for nm23 overexpression, DNA amplification and mutation in aggressive childhood neuroblastomas. *Oncogene* **8**: 855–865

Luo W, Matsuo K, Nagayma Y, Urano T, Furukawa K, Takeshita A, Nakayama Y, Yokoama N, Yamashita S, Izumi M, Shiku H and Nagataki S (1994) Immunohistochemical analysis of expression of nm23H1/nucleoside diphosphate kinase in human thyroid carcinomas: lack of correlation between its expression and lymph node metastasis. *Thyroid* **3**: 105–109

MacDonald NJ, De La Ross A, Benedict MA, Freije JMP, Krutsch H and Steeg PS (1993). A serine phosphorylation of nm23 and not its nucleoside diphosphate kinase activity correlates with suppression of tumour metastatic potential. *J Biol Chem* **268**: 780–789

Mandai M, Konish I, Koshiyama M, Mori T, Arao S, Tashiro H, Okamura H, Nomura H, Hiai H and Fukumoto M (1994) Expression of the metastasis related nm23-H1 and nm23-H2 genes in ovarian carcinomas: correlation with clinicopathology, EGFR, c-erb-B2 and erb-3 genes, and sex steroid receptor expression. *Cancer Res* **54**: 1825–1830

Martinez JA, Prevot S, Nerdlinger B, Nguyen TM, Lacarriere Y, Munier A, Iascu I, Vaillant JC, Capeau J and Lancombe ML (1995) Overexpression of nm23H1 and nm23H2 genes in colorectal carcinomas and loss of nm23H1 expression in advanced tumour stages. *Gut* **37**: 712–720

Nakayama H, Ohtsuru A, Nakao K, Shima M, Nakata K, Watanabe K, Ishi N, Kimura N and Nagataki S (1992) Expression in human hepatocellular carcinoma of nucleoside diphosphate kinase, a homologue of the nm23 gene product. *J Natl Cancer Inst* **84**: 1349–1354

Okabe-Kado J, Kasukabe T, Honma Y, Hayashi M, Henzel WJ and Hozumi M (1992) Identity of a differentiation inhibiting factor for mouse myeloid leukemia cells with nm23/nucleoside diphosphate kinase. *Biochem Biophys Res Commun* **182**: 987–994

Postel EH, Berberich SJ, Flint J and Ferrone CA (1993) Human c-myc transcription factor PuF identified as nm23H2 nucleoside diphosphate kinase a candidate suppressor of tumour metastasis. *Science* **261**: 478–480

Robinson M, Barr L, Fisher C, Fryatt I, Stotter A, Harmer C, Wiltshaw E and Westbury G (1990) Treatment of extremity soft tissue sarcomas with surgery and radiotherapy. *Radiotherapy Oncol* **18**: 221–223

Rosengard AM, Krutzsch HC, Shearn A, Biggs JR, Barker E, Margulies IM, King CR, Liotta LA and Steeg PS (1989) Reduced nm23/AWD protein in tumour metastasis and aberrant Drosophila development. *Nature* **342**: 177–180

Royds JA, Stephenson TJ, Rees RC, Shorthouse AJ and Silcocks PB (1993) nm23 protein expression in ductal in-situ and invasive human breast carcinoma. *J Natl Cancer Inst* **85**: 727–731

Royds JA, Cross SS, Silcocks PB, Scholefield JH, Stephenson TJ and Rees RC (1994) nm23 'anti-metastatic' gene product expression in colorectal carcinoma. *J Pathol* **172**: 261–266

Russell WO, Cohen J, Enzinger F, Hajdu SI, Heise H, Martin RG, Meissner W, Miller WT, Scmitz AL and Suit HD (1977) A clinical staging system for soft tissue sarcomas. *Cancer* **40**: 1562–1570

Stahl JA, Leone A, Rosengard AM, Porter L, King CR and Steeg PS (1991) Identification of a second human nm23 gene, Nm23H2. *Cancer Res* **51**: 445–449

Steeg PS, Bevilacqua G, Kopper L, Thorgeirsson UP, Taimadge JE, Liotta LA and Sobel ME (1988) Evidence for a novel gene associated with low tumour metastatic potential. *J Natl Cancer Inst* **80**: 200–204

© Cancer Research Campaign 1997

Teng DHF, Engele CM and Venkatesh TR (1991) A product of the prune locus of Drosophila is similar to mammalian GTPase activating protein. *Nature* **353**: 437–440

Urano T, Furukawa K and Shiku H (1993) Expression of nm23/NDP kinase proteins on the cell surface. *Oncogene* **8**: 1371–1376

Wallet V, Mutzel R, Troll H, Barzu O, Wurster B, Veron M and Lacombe ML (1990) Dictyostelium nucleoside diphosphate kinase highly homologous to nm23 and AWD proteins involved in mammalian tumour metastasis and Drosophila development. *J Natl Cancer Inst* **82**: 1199–1202

*© Cancer Research Campaign 1997*